# Acquired platinum resistance enhances tumour angiogenesis through angiotensin II type 1 receptor in bladder cancer

**DOI:** 10.1038/bjc.2011.399

**Published:** 2011-10-04

**Authors:** N Tanaka, A Miyajima, T Kosaka, Y Miyazaki, S Shirotake, H Shirakawa, E Kikuchi, M Oya

**Affiliations:** 1 Department of Urology, Keio University School of Medicine, 35 Shinanomachi, Shinjuku-ku, Tokyo 160-8582, Japan

**Keywords:** cisplatin, resistance, angiotensin II type 1 receptor, reactive oxygen species

## Abstract

**Background:**

We investigated the changes in reactive oxygen species (ROS) and angiogenesis through angiotensin II (Ang II) type 1 receptor (AT1R) after the development of acquired platinum resistance in bladder cancer.

**Methods:**

Four invasive human bladder cancer cell lines, T24, 5637, T24PR, and 5637PR, were used *in vitro*, whereas *in vivo*, T24 and T24PR cells were used. T24PR and 5637PR cells were newly established at our institution as acquired platinum-resistant sublines by culturing in cisplatin (CDDP)-containing conditioned medium for 6 months.

**Results:**

Ang II induced significantly higher vascular endothelial growth factor (VEGF) production in T24PR and 5637PR cells than in their corresponding parent cells *in vitro*, whereas Ang II induced a further increase in VEGF production. These platinum-resistant cells also showed significantly higher AT1R expression than their corresponding parent cells. ROS was also significantly upregulated in T24PR and 5637PR cells, whereas increased AT1R expression was significantly downregulated by scavenging free radicals. We also demonstrated the efficacy of AT1R blockade at suppressing the growth of platinum-resistant xenograft model.

**Conclusion:**

Our findings indicate a new molecular mechanism for upregulated AT1R signalling through increased ROS when tumours progressed after the CDDP-based regimens, and shed light on the importance of AT1R blockade for platinum-resistant bladder cancers.

Bladder cancer is one of the most aggressive epithelial tumours and is characterised by a high rate of early systemic dissemination. Patients with metastatic bladder cancer are routinely treated with cisplatin (CDDP)-based systemic chemotherapy, such as M-VAC (methotrexate, vinblastine, doxorubicin, CDDP) and/or GC (gemcitabine, CDDP) regimens. Although CDDP-based regimens still constitute the gold standard, tumours treated with CDDP ultimately acquire platinum resistance and no standard of care exists following CDDP-based regimens.

Angiotensin II (Ang II) is a key biological peptide in the renin-angiotensin system. There are two major subtypes of Ang II receptor: Ang II type 1 receptor (AT1R) and Ang II type 2 receptor (AT2R). Concern regarding the potential role of Ang II in angiogenesis and promotion of tumour growth has been growing (Rivera *et al*, 2001; Egami *et al*, 2003; Juillerat-Jeanneret *et al*, 2004). We previously showed that AT1R signalling led to a potent induction of vascular endothelial growth factor (VEGF) in urogenital cancers (Miyajima *et al*, 2002; Kosugi *et al*, 2006; Kosaka *et al*, 2007, 2010). Moreover, we reported that increased reactive oxygen species (ROS) generation in cancer cells could upregulate AT1R expression and enhance VEGF production in bladder cancer (Tanaka *et al*, 2010).

Several studies have evaluated the potential roles of ROS generation in angiogenesis and tumour growth, and reported that ROS generation could induce the activation of mitogen-activated protein kinase, nuclear factor-*κ*B, activator protein 1, and VEGF production, all of which are strongly associated with tumour development (Schreck *et al*, 1992; Stevenson *et al*, 1994; Puri *et al*, 1995), although the role of ROS generation in cancer cells remains controversial, because it also increases pro-apoptotic molecules such as p53 and p38 mitogen-activated protein kinase (Lee, 1998; Lau *et al*, 2010). To the best of our knowledge, the association between ROS generation and angiogenesis after the development of acquired platinum resistance has not yet been fully elucidated. Moreover, there has been no finding suggesting the regulation of AT1R expression in these tumours.

In the present study, we established acquired platinum-resistant bladder cancer cell lines to evaluate the changes in angiogenesis after the development of acquired platinum resistance. Next, focusing on ROS generation, we investigated the regulation of AT1R expression after the development of acquired platinum resistance, and whether AT1R blockade could suppress the growth of platinum-resistant tumours.

## Materials And Methods

### Reagents

CDDP and paclitaxel were kindly supplied by Nippon Kayaku Co. (Tokyo, Japan), and the ARB olmesartan was kindly provided by Daiichi Sankyo (Tokyo, Japan). Ang II and mouse monoclonal antibody for beta-actin were purchased from Sigma (Atlanta, GA, USA). Gemcitabine, carboplatin, and edaravone (a free radical scavenger) were obtained from Wako Pure Chemical Industries (Osaka, Japan). Mouse monoclonal antibody for CD34 was purchased from Nichirei (Tokyo, Japan). Rabbit polyclonal antibody for VEGF and AT1R were purchased from Santa Cruz Biotechnology, Inc. (Santa Cruz, CA, USA).

### Cell lines and culture

Two invasive human bladder cancer cell lines, T24 and 5637, were obtained from the American Type Culture Collection (Rockville, MD, USA). All cells were routinely maintained in RPMI-1640 (Invitrogen, Carlsbad, CA, USA) supplemented with 10% fetal bovine serum (Dainippon Pharmaceutical, Tokyo, Japan), at 37°C in a humidified 5% CO_2_ atmosphere. To develop platinum resistance, T24 and 5367 cells were grown in RPMI-1640 supplemented with 10% fetal bovine serum containing CDDP, at 37°C in a humidified 5% CO_2_ atmosphere. The concentration of CDDP was increased to 3 *μ*M. These cells were passaged upon reaching confluence over a 6-month period. These new cell lines were named T24PR and 5637PR (acquired platinum resistance for 6 months).

### Murine xenograft bladder cancer model

Six-week old athymic nude BALB/C mice were obtained from Sankyo Lab Service (Tokyo, Japan). T24 and T24PR cells (2 × 10^6^ cells), suspended in 100 *μ*l of matrigel (Becton Dickinson Labware, Lincoln Park, NJ, USA), were implanted subcutaneously into the flank of each mouse, and assigned to each group, which consists of 10 animals. Tumour volume was calculated twice a week using the formula: tumour volume (mm^3^)=length × width × height × 0.52.

To evaluate the efficacy of the ARB olmesartan, on day 14 after cancer cell implantation, the mice were administered olmesartan (10 mg kg^−1^ per day) by gavage. To investigate the sensitivity to CDDP, the mice were administered CDDP (2 or 10 mg kg^−1^) intraperitoneally on day 21 after cancer cell implantation. Paclitaxel (5 or 15 mg kg^−1^) was also administered on day 21 after cancer cell implantation. To evaluate the changes in AT1R expression induced by edaravone, the mice were administered edaravone (5 mg kg^−1^) intraperitoneally daily, beginning on day 14 after cancer cell implantation. All animals were killed on the 28th day, and the subcutaneous tumours were harvested. These experiments were carried out in accordance with the Japanese government guidelines, and the protocol was approved by the Animal Care Committee of our institution.

### Cell growth assay

All cell lines were seeded at a density of 1 × 10^4^ per well into 96-well culture plates. Following 24 h incubation in RPMI 1640 medium with 10% fetal bovine serum, the cells were incubated for the appropriate time with various concentrations of CDDP, paclitaxel, gemcitabine, carboplatin, Ang II, and olmesartan, to investigate the sensitivity of each cell line. At the end of the incubation period, cell viability was determined using a Premix WST-1 Cell Proliferation Assay System (Takara Bio Inc., Shiga, Japan) and microplate spectrophotometer (Bio-Rad Laboratories Inc., Tokyo, Japan).

### Apoptosis assay

Flow cytometric analysis was performed using TUNEL assay to detect apoptosis. TUNEL assay was performed using ApopTag kits (Sigma Chemical). The cells (1 × 10^6^) were seeded in 100-mm dishes, incubated for 24 h in RPMI 1640 medium with 10% fetal bovine serum, and then incubated with various concentrations of CDDP. Following 48 h incubation in medium containing CDDP, apoptosis was detected by flow cytometry, and subsequent analysis was carried out according to the manufacturer's protocol.

### Cell extracts and western blot analysis

Whole-cell extracts were obtained using RIPA buffer (Cell Signaling Technology Japan, Tokyo) containing protease inhibitors, according to the manufacturer's protocol. The extracted whole protein (50 *μ*g) with sample buffer containing 2-mercaptoethanol was separated on 12.5% SDS-PAGE and transferred to a nitrocellulose membrane (Bio-Rad Laboratories, Hercules, CA, USA), and then incubated with 5% skim milk overnight. The following protocols including primary and secondary antibody were described previously (Tanaka *et al*, 2010).

### Fluorescent assay

DCFH-DA, which permeates into cells and interacts with intracellular ROS to generate fluorescent dichlorofluorescein (DCF), was used to measure the intracellular levels of ROS generated. The amount of ROS was estimated from DCF production (Cell Biolabs Inc., San Diego, CA, USA) using fluorescence intensity in the cells at 480 nm excitation per 530 nm emission, using a fluorometric plate reader (Tanaka *et al*, 2010). Glutathione and glutathione S-transferase activity were measured by fluorescent assay using a commercially available detection kit (Anaspec, Fremont, CA, USA).

### Immunostaining for CD34, VEGF, AT1R, and apoptosis

Formalin-fixed paraffin-embedded sections (4 *μ*m) were deparaffinised, rehydrated, and washed in PBS. Endogenous peroxidase was quenched and sections were blocked with skim milk. Primary antibodies of CD34, VEGF, and AT1R were then applied at room temperature for 1 h. After washing with PBS, they were incubated with secondary antibodies against mouse and rabbit IgG conjugated to a peroxidase-labelled dextran polymer for 1 h.

Microvessel density (MVD) in areas of the tumour was counted with anti-CD34 antibody according to the method reported by Kosugi *et al* (2009). The intensity of AT1R and VEGF staining was evaluated according to the method described in our previous report (Tanaka *et al*, 2010). Apoptosis was measured by transferase-mediated nick-end labelling assay using a commercially available apoptosis *in situ* detection kit (Wako Pure Chemical). The apoptosis index was calculated as the average number of transferase-mediated nick-end labelling-positive cells in × 400 fields.

### ELISA assay for VEGF in conditioned medium

Cells were seeded in 60-mm^2^ dishes and allowed to attach at 37 °C in a humidified 5% CO_2_ atmosphere. After 24 h, the medium was replaced with serum-free medium for 4 h. Next, the cells were incubated with conditioned medium. After 20 h, the supernatant was collected and VEGF was measured using a commercially available ELISA kit (Quantikine, R&D Systems, Minneapolis, MN, USA).

### Statistical analysis

All data are presented as the mean±s.e. Statistical analyses were performed using the Mann–Whitney *U*-test. *P*-values <0.05 were accepted as being statistically significant. All statistical analyses were performed using commercially available statistical software.

## Results

### Establishment of the acquired platinum-resistant bladder cancer sublines *in vitro*

Using 5637 and T24 cells, we generated bladder cancer sublines that acquired resistance to platinum, and the cells that were successfully cultured for 6 months in condition medium of CDDP were named 5637PR and T24PR. Following 3 months without CDDP-exposure, further examinations were performed. The doubling times of each PR cell line *in vitro* were slightly increased, compared with their parent cells (34.0±3.7 h in 5637 cells *vs* 37.0±2.5 h in 5637PR cells, 18.6±1.4 h in T24 cells *vs* 21.1±2.2 h in T24PR cells).

5637PR and T24PR cells were treated with various concentrations of CDDP for 48 h (Table 1). CDDP did not strongly affect the viability of 5637PR and T24PR cells, compared with their corresponding parent cells. Using the TUNEL assay, we also investigated the apoptosis induced by CDDP. The apoptotic index induced by CDDP (20 *μ*M) at 48 h was 36.4±12.8% in 5637 cells, 2.3±0.6% in 5637PR cells, 41.5±8.7% in T24 cells, and 2.3±1.0% in T24PR cells. These results were correlated with the CDDP-induced cell viability of each cell line. Although no significant difference was observed in the level of glutathione, both 5637PR (1.2-fold) and T24PR (1.3-fold) cells showed slightly higher levels of glutathione S-transferase activity, compared with that of the corresponding parent cells (*P*<0.05). We then evaluated the changes in sensitivity to other anticancer agents including paclitaxel, gemcitabine, and carboplatin, using a 48-h continuous exposure (Table 1). Under these experimental conditions, both 5637PR and T24PR cells showed cross-resistance to all anticancer agents, whereas the sensitivity of paclitaxel did not change dramatically, compared with the other agents.

### Establishment of the acquired platinum-resistant bladder cancer subline *in vivo*

**Figure 1:**
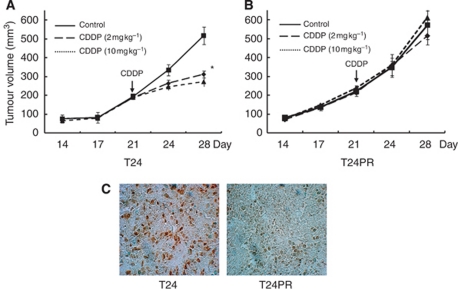
Changes in cell viability and apoptosis induced by CDDP before and after the development of acquired platinum resistance *in vivo*. For *in vivo* study, CDDP (2 or 10 mg kg^−1^) was injected intraperitoneally on the 21st day. ^*^P<0.05, compared with control group. Each value represents the mean±s.e. (**A**) Time course changes in tumour growth in T24 bladder cancer xenograft models treated with CDDP. (**B**) Time course changes in tumour growth in T24PR bladder cancer xenograft models treated with CDDP. (**C**) Serial photo panels showing TUNEL staining of T24 and T24PR mouse xenograft tumours treated with CDDP (10 mg kg^−1^) on the 28th day. Magnification is 1 : 400.

To evaluate the acquired platinum resistance *in vivo*, T24 and T24PR tumours were administered CDDP (2 or 10 mg kg^−1^; Figures 1A and B). Although CDDP significantly suppressed tumour growth in T24 cells even at the low dose (2 mg kg^−1^), CDDP did not significantly suppress tumour growth in T24PR cells even at high dose (10 mg kg^−1^). The apoptotic index of T24PR tumours on the 28th day (1.67±0.21, *P*<0.05) was significantly lower than that of T24 tumours (10.8±0.60) at the high dose of CDDP (Figure 1C). Similar results were also observed at the low dose.

### Enhancement of VEGF production through upregulation of AT1R signalling in platinum-resistant bladder cancer sublines

**Figure 2:**
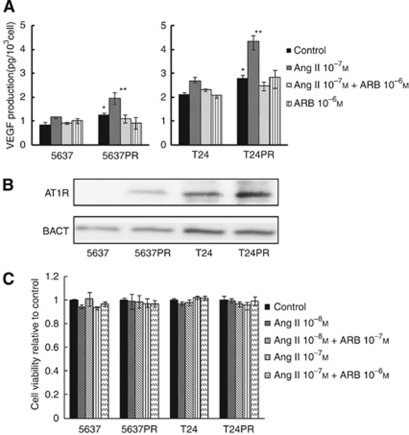
Changes in VEGF production induced by Ang II and AT1R expression before and after the development of acquired platinum resistance. Each value represents the mean±s.e. of at least three individual experiments. (**A**) VEGF measurements in four bladder cancer cell lines after 20-h incubation with and/or without Ang II and olmesartan. ^*^P<0.05, compared with corresponding parent cells without Ang II stimulation. ^**^P<0.05, compared with corresponding parent cells with Ang II stimulation. (**B**) AT1R expression in four bladder cancer cell lines. (**C**) Effect of Ang II and olmesartan on four bladder cancer cell lines for 48 h.

We examined the changes in VEGF production induced by Ang II. The cells were exposed to Ang II (10^−7^ M) with or without olmesartan (10^−7^ M), and VEGF in the supernatant was assessed by ELISA (Figure 2A). Ang II induced a further increase of VEGF production in platinum-resistant cell lines compared with those of parent cells, whereas olmesartan significantly inhibited Ang II-induced VEGF production.

Next, we examined AT1R expression in each cell line (Figure 2B). Using Western blot analysis, both 5637PR and T24PR showed significantly higher AT1R expression than their corresponding parent cells. To examine the Ang II-inducing effects on cell growth, 5637PR and T24PR cells were incubated with and without Ang II and olmesartan for 48 h (Figure 2C). *In vitro* proliferation assay at clinically achievable concentrations of Ang II (10^−8^ to 10^−7^ M) and olmesartan (10^−7^ to 10^−6^ M) showed no effects on cell proliferation. Similar results were observed at 24 and 72 h.

### Upregulation of ROS generation in platinum-resistant bladder cancer sublines and the free radical scavenger edaravone downregulates the expression of AT1R

**Figure 3:**
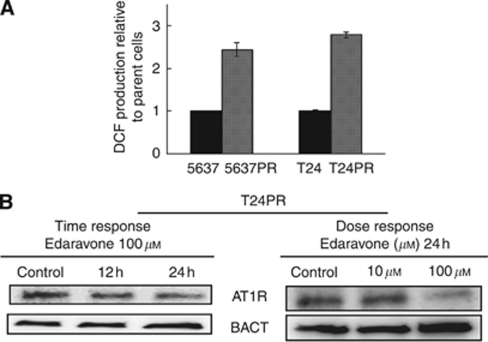
Changes in ROS generation before and after the development of acquired platinum resistance, and the regulation of AT1R expression induced by the free radical scavenger edaravone in acquired platinum-resistant cells. Each value represents the mean±s.e. of at least three individual experiments. (**A**) ROS generation in four bladder cancer cells was measured by DCF production. (**B**) Western blotting showed AT1R expression was downregulated, using edaravone in a time- and dose-dependent manner.

To elucidate the mechanism for upregulating AT1R expression, we investigated ROS generation by measuring DCF production. ROS generation in the platinum-resistant sublines was significantly higher than that of their respective parent cells (Figure 3A). Next, to examine whether increased ROS generation after development of the acquired platinum resistance could affect AT1R expression, we used the free radical scavenger edaravone. To evaluate the edaravone-induced effects on cell growth, T24PR cells were incubated with various concentrations of edaravone. Edaravone showed no effects on cell proliferation for 24 h.

Next, AT1R expression in T24PR cells was evaluated after treating with edaravone, and AT1R expression was significantly downregulated in a time- and dose-dependent manner (Figure 3B). We then evaluated AT1R expression after treating with edaravone *in vivo*. The results of immunostaining indicated that AT1R expression was significantly suppressed in edaravone-treated tumours (2.5±0.2 in the control group *vs* 1.1±0.2 in the edaravone group, *P*<0.05), and these results were consistent with those in the *in vitro* study.

### Efficacy of ARB administration in a murine xenograft model of platinum-resistant bladder cancer

**Figure 4:**
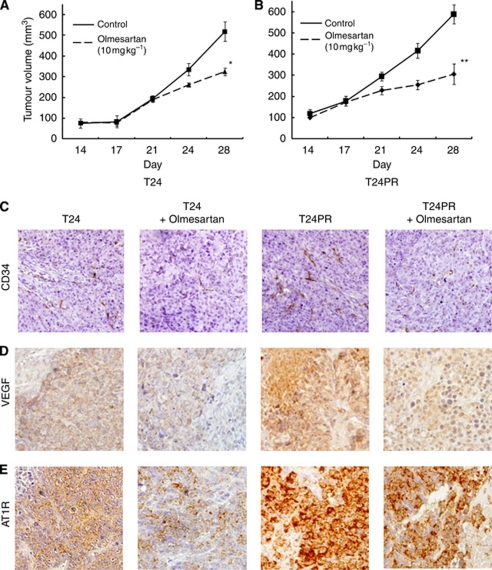
Time course changes in tumour growth in T24 bladder cancer xenograft models (**A**), and that of T24PR bladder cancer xenograft models treated with olmesartan (**B**). Olmesartan (10 mg kg^−1^ per day) was started on the 14th day. ^*^P<0.05, compared with control of T24 tumours. ^**^P<0.05, compared with control of T24PR tumours. Serial photo panels showing immunohistochemical staining of CD34 (**C**), VEGF (**D**), or AT1R expression (**E**), in T24 and T24PR tumours with or without olmesartan on the 28th day. Magnification is 1 : 400.

We examined the efficacy of ARB in T24PR tumours. As shown in Figure 4A, olmesartan (10 mg kg^−1^) administered daily by gavage significantly suppressed the growth of T24 tumours, and tumour volume was 63.0% compared with the control group on the 28th day. Moreover, olmesartan (10 mg kg^−1^) also significantly suppressed the growth of T24PR tumours (Figure 4B). The tumour volume was decreased to 51.9% compared with the control group on the 28th day. Significant differences in tumour volume were observed between the ARB-treated group and control group as early as day 24 after tumour implantation.

### Changes in MVD, VEGF, and AT1R expression after ARB administration in murine xenograft model of platinum-resistant bladder cancer

We examined angiogenic parameters using immunohistochemical techniques on the 28th day (Figures 4C and D). The MVD of T24PR tumours (7.8±0.8, *P*<0.05) was significantly higher than that of T24 (4.3±0.6). T24PR tumours showed significantly higher VEGF expression (2.5±0.3, *P*<0.05) than T24 tumours (1.5±0.2), whereas T24PR tumours also showed significantly higher AT1R expression (2.5±0.2, *P*<0.05) than T24 tumours (1.5±0.3). These results suggest that neovascularisation is the more aggressively induced platinum-resistant tumours, and were consistent with those of AT1R expression.

We then investigated MVD, VEGF, and AT1R expression after ARB treatment. VEGF expression in T24PR tumours was significantly suppressed by olmesartan (1.3±0.1, *P*<0.05), whereas the MVD of T24PR tumours was also decreased in the olmesartan group (3.5±0.2, *P*<0.05). In addition, olmesartan administration affected the level of AT1R expression (2.0±0.1, *P*<0.05). Similarly, the expressions of MVD, VEGF, and AT1R in T24 tumours were also significantly decreased in the olmesartan-treated group. Therefore, AT1R blockade might be effective even after the tumours developed acquired platinum resistance.

### Effect of combination therapy with paclitaxel and ARB in murine xenograft model of platinum-resistant bladder cancer

**Figure 5:**
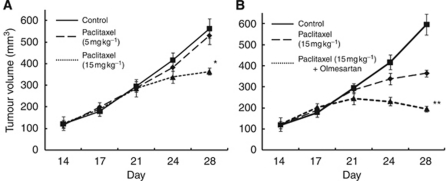
Time course changes in tumour growth in T24PR bladder cancer xenograft models treated with paclitaxel only (**A**). Paclitaxel (5 or 15 mg kg^−1^) was injected intraperitoneally on the 21st day. ^*^P<0.05, compared with control group. Time course changes in tumour growth in T24PR bladder cancer xenograft models treated with paclitaxel and olmesartan (**B**). Olmesartan (10 mg kg^−1^ per day) was started on the 14th day, and paclitaxel (15 mg kg^−1^) was injected intraperitoneally on the 21st day. ^**^P<0.05, compared with paclitaxel-only group.

Although results in a WST-1 assay indicated the sensitivity of paclitaxel did not change dramatically, compared with other agents, we evaluated the efficacy of paclitaxel monotherapy in T24PR tumours. As shown in Figure 5A, paclitaxel (5 or 15 mg kg^−1^) was administered on day 21 after cancer cell implantation, and tumour growth was significantly suppressed in a dose-dependent manner. We then investigated the efficacy of the combination of paclitaxel and ARB. As shown in Figure 5B, olmesartan (10 mg kg^−1^) was administered daily by gavage from day 14 after cancer cell implantation, and the tumour growth was significantly suppressed in paclitaxel+ARB-treated tumours, compared with paclitaxel only (*P*<0.05).

## Discussion

In the present study, we analysed two different molecular events that occurred after the development of acquired platinum resistance in bladder cancer. First, ROS generation induced by cancer cells was significantly increased after the development of acquired platinum resistance, and second, AT1R expression in these cells was significantly upregulated through increased ROS generation, resulting in more angiogenic aggressiveness. On the basis of these molecular mechanisms, we also showed the efficacy of AT1R blockade against platinum-resistant tumours. To the best of our knowledge, this is the first report that shows a correlation between increased ROS generation and AT1R expression after the development of acquired platinum resistance, and the significance of AT1R blockade as a new modality for platinum-resistant bladder cancer.

The prognosis for patients with advanced or metastatic bladder cancer remains poor. The vast majority of patients treated with CDDP-based regimens develop progressive disease within 8 months of treatment, and the median survival is reported to be only 13–15 months (von der Maase *et al*, 2000). There currently is still no approved treatment option for patients who develop disease recurrence or progression after CDDP-based regimens.

Increased ROS generation in cancer cells correlates with their tumour aggressiveness and poor prognosis, and previous reports have suggested that the increased ROS generation in cancer cells has a pivotal role in the acquisition of the hallmarks of cancer (Patel *et al*, 2007; Kumar *et al*, 2008). In contrast, if the increase of ROS generation reaches a certain threshold level that is incompatible with cellular survival, ROS generation may exert a cytotoxic effect, leading to the death of cancer cells and thus limiting tumour progression (Wu, 2006; Fruehauf and Meyskens, 2007). Taken together, ROS generation may work as a double-edged sword, and this paradoxical effect is particularly interesting in terms of how cancer cells can gain growth and survival benefits under intrinsic oxidative stress.

To investigate the association between ROS generation and AT1R expression after the development of acquired platinum resistance, we selected two different cell lines: 5637, which is comparatively resistant to CDDP and has a low level of AT1R, and T24, which is sensitive to CDDP and has a high level of AT1R. We examined the changes in VEGF production induced by Ang II and AT1R expression. *In vitro* study showed that Ang II induced further increases in VEGF production in both the platinum-resistant cells, compared with those of their parent cells. Similarly, both platinum-resistant cells exhibited significantly higher levels of AT1R expression than their parent cells. These results suggest that long-term exposure to CDDP might affect AT1R expression even if the tumours do not show a high level of AT1R expression before CDDP-treatment.

We then examined the changes in ROS generation. ROS generation induced by cancer cells was significantly upregulated in 5637PR and T24PR cells compared with their corresponding parent cells, and were correlated with the changes of AT1R expression. Moreover, we also showed that suppressing ROS generation could significantly induce the downregulation of AT1R expression in acquired platinum-resistant cancer cells. These results suggest a possible molecular mechanism for tumour aggressiveness after the development of acquired platinum resistance that may originate in upregulating the AT1R expression induced by increased ROS generation.

New agents with improved efficacy and adequate tolerability would be needed for patients with advanced bladder cancer who have previously received a CDDP-based regimen. Treatment for these patients is further complicated by poor performance status, comorbidities, and inadequate renal function.

Although angiogenesis in cancer cells is regulated by many mechanisms, it may be controversial as to whether the AT1R pathway is a major regulator of VEGF production, or whether other mechanisms exist. The present study did not fully elucidate whether upregulated AT1R expression could affect the sensitivity to ARBs. However, our results strongly suggest that ARB olmesartan could suppress tumour growth even in the acquired platinum-resistant tumours.

Previous studies also reported the efficacy of AT1R blockade in cancer treatments. Wilop *et al* (2009) analysed retrospectively 287 patients with advanced non-small cell lung cancer undergoing first-line platinum-based chemotherapy, and reported that patients who received angiotensin-converting enzyme inhibitors or ARBs had a longer survival than non-recipients. Nakai *et al* (2010) reported that the use of angiotensin-converting enzyme inhibitors or ARBs with gemcitabine was an independent prognostic factor for both progression-free survival and overall survival in patients with advanced pancreatic cancer. Two prospective studies also proposed the potency of AT1R blockade in cancer treatment (Yoshiji *et al*, 2009; Tatokoro *et al*, 2011). Although an increasing body of evidence suggests the efficacy of ARBs may be promising, we examined the effect of combination therapy consisting of CDDP and olmesartan in T24PR tumours, and found no significant difference in tumour growth between the ARB-only group and CDDP+ARB-treated group (data not shown). Therefore, administration of an ARB may not change the sensitivity to CDDP once a tumour has developed acquired platinum resistance. On the other hand, as shown in Figure 5, although the sensitivity to paclitaxel did not change dramatically compared with other agents, the use of combination therapy with taxane agents (paclitaxel or docetaxel) and ARBs may yield promising results in patients with acquired platinum-resistant tumours.

In summary, acquired platinum resistance may induce more angiogenic aggressiveness through upregulating AT1R expression induced by increased ROS generation in bladder cancer. These findings suggest a new molecular mechanism for AT1R signalling when tumours progress after CDDP-based regimens. As ARBs, which are antihypertensive agents, are already in clinical use without severe side effects, from a clinical point of view, we propose they may be an effective choice in patients who develop disease recurrence or progression after CDDP-treatment.

## Figures and Tables

**Table 1:** Comparison of drug resistance to anticancer agents in 5637, 5637PR, T24, and T24PR cells

	**Cisplatin**		**Paclitaxel**		**Gemcitabine**		**Carboplatin**	
**Cell line**	**IC_50_±s.e. (*μ*M)**	**RF**	**IC_50_±s.e. (nM)**	**RF**	**IC_50_±s.e. (nM)**	**RF**	**IC_50_±s.e. (*μ*M)**	**RF**
5637	4.2±0.1		322±10		508±12		64±2.1	
5637PR	13.5±0.8	3.2	687±25	2.1	2324±33	4.6	210±10	3.3
								
T24	3.8±0.3		44±1.0		165±10		65±4.5	
T24PR	20.2±1.2	5.3	73±2.4	1.7	701±52	4.2	211±11	3.2

Abbreviation: RF=resistance factor.

IC_50_ values were determined in three independent experiments. RF=IC_50_ of resistant line/IC_50_ of parent line.
